# THE TRANSFORMATION OF LATE-LIFE SINGLEHOOD: EMERGENCE OF THE SOCIETY OF DIVORCEES

**DOI:** 10.1093/geroni/igz038.2302

**Published:** 2019-11-08

**Authors:** Torbjorn Bildtgard, Peter Öberg

**Affiliations:** 1 Stockholm University, Stockholm, Sweden; 2 University of Gävle, Gävle, Sweden

## Abstract

More than four decades ago Lopata coined the concept “society of widows” to describe the gendered reality of late life singlehood, where widowed women were excluded from coupled social life and had to depend on other widows for social integration. We have coined the concept “society of divorcees” to describe the changing reality of late life singlehood. Swedish, American and EU census data and a national survey to Swedes 60-90 years old (n=1225; response rate 42%). Results show that more people enter later life as divorcees or become divorced at a high age. Among Swedes 60+ divorcees outnumber widowed people, and the incidence of late life divorce has more than doubled since the millennium in what has been called the grey divorce revolution. Many other Western countries follow the same demographic trend, posing important questions about late life singlehood. Based on two Swedish studies we will show that the structure of the late life single community is becoming less gender skewed as a consequence of the emerging society of divorcees, and that in this society relationship careers are increasingly complex, attitudes to repartnering increasingly liberal and partner sanctification seldom an issue. We conclude by discussing the consequences of the emerging society of divorcees for late life support structures.

